# Direct Observation of Enhanced Nitric Oxide in a Murine Model of Diabetic Nephropathy

**DOI:** 10.1371/journal.pone.0170065

**Published:** 2017-01-19

**Authors:** Margien G. S. Boels, Ernst E. H. van Faassen, M. Cristina Avramut, Johan van der Vlag, Bernard M. van den Berg, Ton J. Rabelink

**Affiliations:** 1 Einthoven Laboratory for Experimental Vascular Medicine, Department of Internal Medicine (Nephrology), Leiden University Medical Center, Leiden, The Netherlands; 2 Department of Molecular Cell Biology, Leiden University Medical Center, Leiden, The Netherlands; 3 Department of Nephrology, Radboud University Medical Center, Nijmegen, The Netherlands; University of Louisville, UNITED STATES

## Abstract

Uncoupling of nitric oxide synthase (NOS) secondary to redox signaling is a central mechanism in endothelial and macrophage activation. To date studies on the production of nitric oxide (NO) during the development of diabetic complications show paradoxical results. We previously showed that recoupling eNOS by increasing the eNOS cofactor tetrahydrobiopterin (BH_4_) could restore endothelial function and prevent kidney injury in experimental kidney transplantation. Here, we employed a diabetic mouse model to investigate the effects of diabetes on renal tissue NO bioavailability. For this, we used *in vivo* NO trapping, followed by electron paramagnetic resonance spectroscopy. In addition, we investigated whether coupling of NOS by supplying the cofactor BH_4_ could restore glomerular endothelial barrier function. Our data show that overall NO availability at the tissue level is not reduced sixteen weeks after the induction of diabetes in apoE knockout mice, despite the presence of factors that cause endothelial dysfunction, and the presence of the endogenous NOS inhibitor ADMA. Targeting uncoupled NOS with the BH_4_ precursor sepiapterin further increases NO availability, but did not modify renal glomerular injury. Notably, glomerular heparanase activity as a driver for loss of glomerular barrier function was not reduced, pointing towards NOS-independent mechanisms. This was confirmed by unaltered increased glomerular presence of cathepsin L, the protease that activates heparanase.

## Introduction

Endothelial dysfunction is assumed to contribute to kidney disease progression in diabetes [1] with endothelial nitric oxide (NO) production as a key feature of healthy endothelium. Uncoupling of NOS secondary to redox signaling is a central mechanism in endothelial activation. We previously showed that recoupling eNOS by increasing the NOS cofactor tetrahydrobiopterin (BH_4_) could restore endothelial function and prevent kidney injury in experimental kidney transplantation [2, 3]. BH_4_ was also shown to modulate the iNOS isoform in macrophages, counterbalancing the redox effector pathway in these cells [4], where macrophage activation has also been implicated in progression of diabetic nephropathy [5, 6].

Conflicting results about the role of NO in diabetic nephropathy have, however, been publish *in vitro* as well as *in vivo*. Enhanced NO generation have been reported in studies of cultured hyperglycemic endothelial cells, advanced diabetes and diabetic nephropathy [7–9], whereas NO availability has also been reported to be diminished in the setting of human [10–12] and rodent [13–15] diabetic nephropathy. Also a time dependent effect has been reported: NO generation during the early stages of nephropathy was increased while it decreased in later stages [16, 17].

While eNOS knockout mice have proved to be invaluable for studies assessing the consequences of reduced NO availability in diabetic nephropathy [18], the absence of NO make these mice unsuited for our studies. Therefore, we treated apolipoprotein E knockout mice (apoE KO) with streptozotocin to induce stable and reproducible diabetic nephropathy [19, 20]. We used *in vivo* NO spin-trapping with iron-dithiocarbamate complexes, a highly-sensitive quantitative technique that enables one to detect localized concentrations of trapped NO *in vivo* [21–24]. Also, we assessed glomerular endothelial function, by determining glycocalyx integrity and barrier function.

Surprisingly, we observed enhanced renal NO bioavailability in the setting of diabetic nephropathy. We also found that sepiapterin, a BH_4_ precursor, while further augmenting local NO levels, did not modify renal glomerular injury. This suggests that diabetes-induced endothelial dysfunction is not directly associated with renal NO deficiency. Notably, glomerular heparanase activity as a driver for loss of glomerular barrier function was not reduced, pointing towards NOS-independent mechanisms.

## Materials and Methods

### Diabetic ApoE KO mouse model

**Fig 1:**
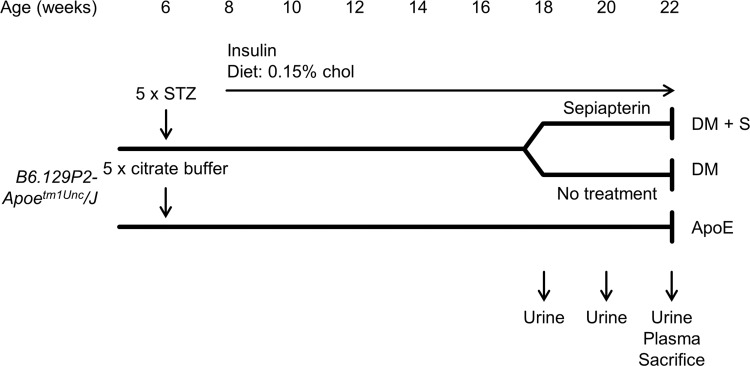
Experimental set-up for assessment of NO bioavailability. (A) male ApoE KO mice (*B6*.*129P2- Apoe*^*tm1Unc*^*/J)* were injected with citrate buffer ± STZ. Diabetic mice received cholesterol enriched diet and insulin from week 8 onwards. At 18 week of age, diabetic mice were treated with sepiapterin or received normal drinking water for 4 weeks. Urine was collected upon commencing with the experimental procedure and after 2 and 4 weeks of treatment. At 22 weeks, plasma was collected and the mice were sacrificed.

All animal experiments were approved by the ethical committee on animal care and experimentation of the Leiden University Medical Centre. All animal work was performed in compliance with the Dutch governmental guidelines. For our experiments, we used three groups of six weeks old male ApoE KO mice (Jackson Laboratory, Bar Harbor, ME). Diabetes was induced in two groups by intraperitoneal injections with streptozotocin (STZ, 60 mg/kg; Sigma-Aldrich, St. Louis, MO) in citrate buffer (0.1 mol/L, pH = 4.5) for five consecutive days, according to the DiaComp protocol (Fig 1). The non-diabetic control group was injected with citrate buffer alone. All mice were housed under normal day-night cycle with free access to drinking water and chow. Non-diabetic apoE KO mice received standard chow, whereas diabetic apoE KO mice received cholesterol-enriched chow (0.15 wt%) (Technilab-BMI, Someren, The Netherlands). Twelve weeks after the induction of diabetes, these mice were divided in two groups: one group was treated with sepiapterin (10 mg/kg daily) in drinking water for four consecutive weeks, to increase NO levels. The other group received normal drinking water. Data obtained from non-diabetic apoE KO mice as well as control diabetic apoE KO mice are previously published [19].

Blood glucose concentrations were measured with an Accu-check glucose meter (Roche, Basel, Switzerland). When glucose concentrations exceeded 25 mmol/L, mice were treated with 1–2 units insulin (Lantus, Aventis Pharmaceuticals, Bridgewater, NJ, US) up to three times per week. Sixteen weeks after STZ injections, at 22 weeks of age, mice were sacrificed to perform NO-trapping in kidney, liver and heart.

### Urine collection and analyses

A 24-hour urine sample was collected at 12, 14 and 16 weeks after STZ injections. Urinary albumin and creatinine concentrations were determined as previously described [19]. In brief, albumin concentrations were quantified with Rocket immunoelectrophoresis [25] and creatinine concentrations were quantified by the Jaffé method using 0.13% picric acid and a creatinine standard set (Sigma-Aldrich).

### Immunohistochemistry

Following sacrificing by CO_2_ asphyxiation, one half of a kidney was used for histological assessment of classical markers of glomerular damage and morphometric changes to confirm diabetic nephropathy. For this, capillary and mesangial matrix area were quantified in Periodic acid-Schiff (PAS) or Trichrome-stained paraffin embedded 4 μm sections, respectively. We used a semiautomatic image analyzing system (Leica Q600 Qwin; Leica Microsystems, Cambridge, UK) to determine the fraction of glomerular surface area by the point-counting method.

Glomerular heparanase expression was quantified after overnight incubation with primary antibody (Polyclonal rabbit anti-heparanase 1.5 μg/mL, InSight Biopharmaceuticals, Rehovot, Israel), followed by goat anti-rabbit IgG-Alexa 594 (1/1000), for 1 hour, both in blocking buffer. Sections were counterstained with Hoechst (1/1000) and embedded in Vectashield mounting medium (Vector Laboratories Inc., Burlingame, CA). Cathepsin L polyclonal antibody (R&D Systems) was incubated overnight, followed by horseradish peroxidase–conjugated secondary antibody and 3,3’-diaminobenzidine and counterstained with hematoxylin. Staining area was quantified as the percentage of stained area divided by the glomerular area.

### Determination of nitric oxide

In all three groups, endogenous NO bioavailability was measured by *in vivo* spin trapping with iron-diethyldithiocarbamates (Fe^2+^-DETC) complexes as previously described [19]. After 30 minutes of spin-trapping, mice were sacrificed using CO_2_ asphyxiation. Subsequently, ~350 mg sections of kidney, liver and heart tissues were submerged in HEPES buffer (450 μl, 150 mmol/L, pH 7.4) and snap frozen with liquid nitrogen. The yield of paramagnetic ferrous mononitrosyl-iron complexes (MNIC) was determined with electron paramagnetic resonance (EPR) spectroscopy. For this, frozen tissue samples were measured at 77 K with an X-band EMX-Plus spectrometer (Bruker BioSpin, Rheinstetten, Germany) equipped with a Bruker liquid finger Dewar flask filled with liquid nitrogen. Spectrometer settings were microwave power, 20 mW; time constant, 82 ms; analog-to-digital conversion time, 82 ms; and detector gain, 10^4^. The magnetic field was modulated with 5-G amplitude at a frequency of 100 kHz. With these settings, a single field sweep provided adequate sensitivity. During the experiments, the inside of the EPR cavity (ER 4119 HS-W1, cylindrical TE_011_ mode; Bruker) was continuously flushed with dry nitrogen to prevent condensation of ambient humidity on the cool Dewar flask.

### Determination of plasma ADMA concentrations

Asymmetric N^G^, N^G^-dimethyl-L-arginine (ADMA) is a endogenous inhibitor of nitric oxide synthases. Therefore, we measured plasma ADMA concentrations with a commercially available enzyme-linked immunosorbent assay kit (DLD Diagnostika GmbH, Hamburg, Germany) according to the manufacturer's protocol.

### Determination of glomerular endothelial glycocalyx coverage

For electron microscopic visualization of the glycocalyx, three mice per group were anesthetized (intraperitoneal). Left kidneys were perfused with 0.5% bovine serum albumin (BSA) and 5 U/mL heparin in 5 mL Hepes-buffered salt solution (HBSS), followed by 2 mL of cationic ferritin (horse spleen, 2.5 mg/mL, Electron Microscopy Sciences, Fort Washington, PA) in HBSS alone at 2 mL/minute, as described before [19]. Kidneys were excised, the capsules removed and stored in fixative (1.5% glutaraldehyde + 1% paraformaldehyde in 0.1 mol/L sodium-cacodylate buffered solution, pH 7.4) overnight at 4°C and further prepared for transmission electron microscopy (TEM). Data was collected into large virtual slides (stitches) that provide an overview of the glomeruli, allowing for high-detail assessment and quantitative analysis of glomerular (patho)physiology [26]. Images (2Kx2K) were acquired at an acceleration voltage of 120 kV, on FEI Tecnai T12 microscopes (FEI, Eindhoven, the Netherlands), equipped with FEI Eagle 4Kx4K CCD cameras. The polyanionic glycocalyx on the surface of endothelial cells can be visualized using TEM by binding of electron-dense cationic substances, such as cationic ferritin [27]. Within the stitches, individual capillary loops were captured and glycocalyx coverage was quantified in 6–11 capillary loops in 9 glomeruli. The percentage of cationic ferritin positive endothelial surface coverage was determined using an automatic grid overlay in the public domain NIH ImageJ version 1.46. For every capillary, a minimum of 80 crosshairs was at the intersection of the endothelium. This resulted in a percentage glycocalyx positive area.

### Statistical analysis

Data are presented as mean ± SD, unless stated otherwise. Changes in ACR during treatment were analyzed using a linear-mixed model regression analysis, since samples were collected over time and were therefore animal dependent (SPSS Statistics, version 20, IBM). Differences in other experiments with continuous variables were determined using one-way ANOVA and post hoc analysis with Tukey’s multiple comparison test. P<0.05 was considered statistically significant.

## Results

### Diabetes increases renal NO levels

**Fig 2:**
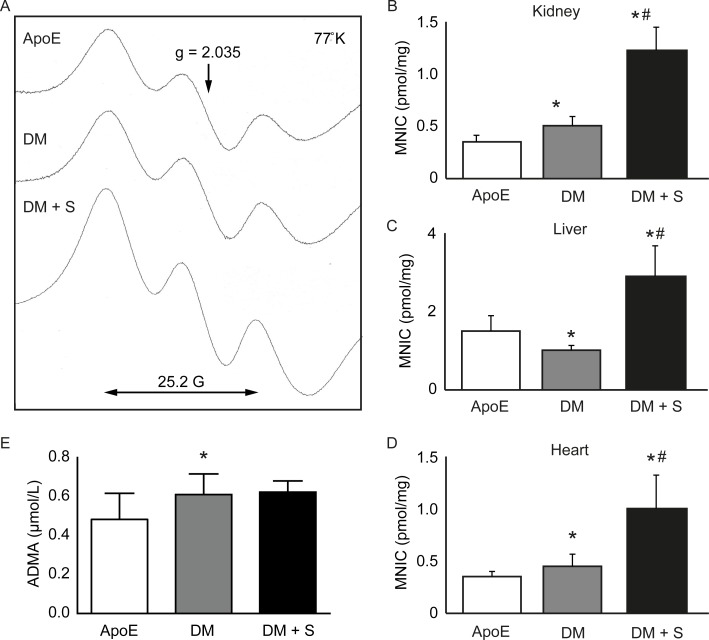
Tissue-dependent variation in NO free radical induction. (A) EPR spectrum of frozen kidney samples. The characteristic triplet structure of the mononitrosyl-iron complex (MNIC, double-headed arrow) centers around g  =  2.035 and represents the formation of local nitric oxide in 334–370 mg tissue. (B-D) Quantification of nitric oxide formation in kidney, liver and heart tissue, shown as mean pmol MNIC / mg wet tissue ± SD, n = 7–9. E) Plasma ADMA concentrations, shows as mean ± SD, n = 8. *P<0.05, compared with ApoE; #P<0.05 compared with DM. ApoE = ApoE KO mice, DM = diabetic apoE KO mice, DM + S = diabetic apoE KO mice + sepiapterin.

NO levels were determined as MNIC yields in various tissues (Fig 2), showing considerable variation between the tissue types. In particular, the liver was found to produce higher quantities of NO compared to renal and cardiac tissue. The findings in non-diabetic apoE KO mice are in qualitative concordance with previous findings in other rodent models such as rats [28] and C57Bl/6 mice (the latter revealing 0.34 ± 0.1 pmol MNIC / mg in kidney; 2.1 ± 1.0 in liver and 0.52 ± 0.1 pmol MNIC / mg heart). Upon STZ-induced diabetes, apoE KO mice revealed increased renal NO (0.51 ± 0.16 in diabetic apoE KO mice vs 0.29 ± 0.12 pmol MNIC / mg tissue in non-diabetic apoE KO, P<0.01 [19]). Of note, the effect of diabetes on NO bioavailability in cardiac tissue was considerably smaller (P<0.02), whereas STZ induced a reduction in NO bioavailability in hepatic tissue (P<0.01). Clearly, STZ-induced diabetes affects NO homeostasis in a tissue-dependent fashion, although prolonged treatment of diabetic mice with sepiapterin, a tetrahydrobiopterin (BH_4_) precursor, was found to induce a 2- to 3-fold increase of bioavailable NO in kidney, heart and liver tissue (Fig 2).

To see how these different observations at the tissue level reflect those observed in plasma, we also determined the formation of asymmetric dimethyl arginine (ADMA), a known endogenous inhibitor of eNOS. These studies uncovered a small increase in plasma ADMA concentrations upon STZ-induced diabetes (0.48 ± 0.13 μmol/L vs. 0.61 ±0.11 μmol/L, P<0.05; Fig 2E). Treatment with sepiapterin for four weeks did not affect plasma ADMA concentrations.

### Increased NO levels do not improve diabetic nephropathy in diabetic apoE KO mice

**Fig 3:**
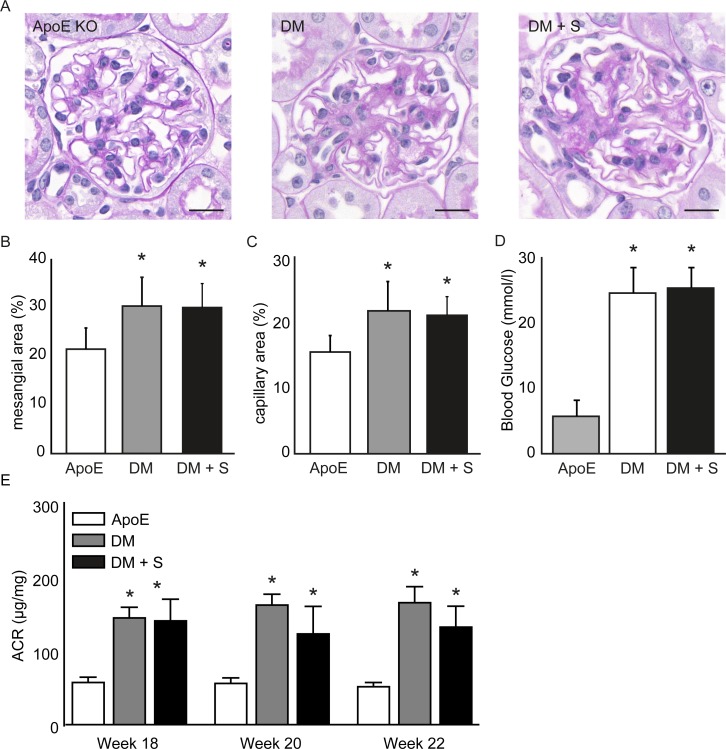
Sepiapterin does not reduce albuminuria in diabetic apoE KO mice. (A) PAS-stained glomeruli of apoE KO mice (apoE), diabetic apoE KO mice (DM) and diabetic apoE KO mice treated with sepiapterin (DM + S), showing heterogeneous diabetic lesions 14 weeks after induction of diabetes with STZ (20). Scale bars: 20 μm. Sepiapterin did not affect mesangial area (B,C), nor blood glucose concentrations (D). Data are shown as mean ± SD, *P<0.05 compared with apoE, n = 8. (E) Albumin-creatinine ratios (ACR) at baseline, 2- and 4 weeks after treatment, as indicated by mean ± SEM, *P<0.05 compared with apoE, n = 14–23.

Fourteen weeks after inducing diabetes, we observed common characteristics of diabetic nephropathy, including mesangial expansion, mesangiolysis, and, glomerular hypertrophy, as we described elsewhere [19]. An ensuing treatment with sepiapterin for four weeks did not prevent the development of diabetic renal lesions as quantified in PAS- and Trichrome-stained glomeruli (Fig 3A–3C). Importantly, for these studies we verified that sepiapterin did not affect blood glucose concentrations at the selected dose (Fig 3D).

To assess the effect of sepiapterin on kidney function, we collected 24-hours urine samples prior to treatment with sepiapterin, as well as at 2 and 4 weeks after treatment. Diabetic apoE KO mice were characterized by progressive albuminuria (Fig 3E [19]), which is in keeping with a parallel increase in urine production and urinary albumin excretion [19]. Multiple comparisons reveal that a 4-week sepiapterin treatment regimen is insufficient to reduce albuminuria as compared to non-treated mice (-11.8 ± 7.1%, p = 0.46).

**Fig 4:**
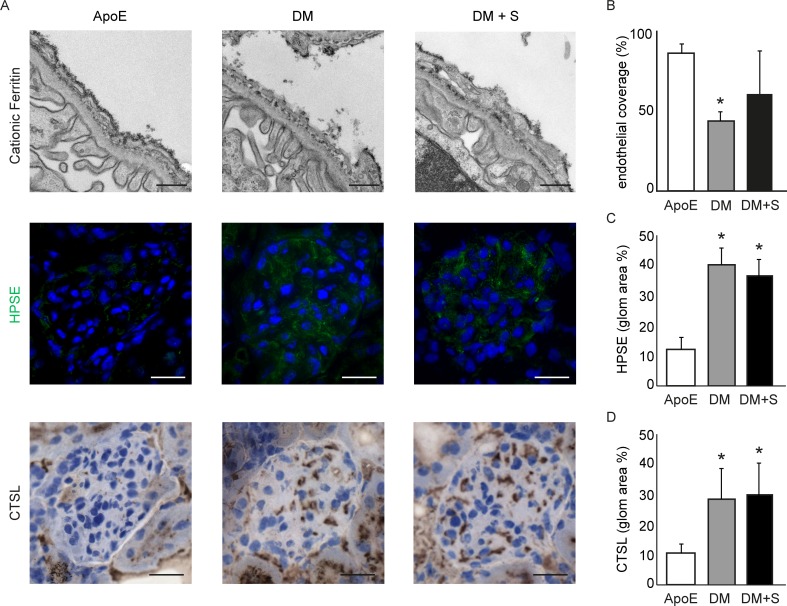
Increased NO levels affect endothelial glycocalyx non-uniformly. (A) Representative microscopic images of cationic ferritin (TEM; top), heparanase (HPSE, immunofluorescence; middle) and cathepsin L (CTSL; bottom) in glomeruli of apoE KO mice (apoE), diabetic apoE KO mice (DM) and diabetic apoE KO mice treated with sepiapterin (DM + S). (B) Quantification of endothelial cationic ferritin coverage in 6–8 capillary loops in 9 glomeruli of 3 mice, shown as mean percentage of total capillary length ± SD, (C) Quantification of glomerular heparanase expression, shown as mean area percentage ± SD. (D) Quantification of glomerular cathepsin L expression, shown as mean area percentage ± SD. *P<0.05 compared with ApoE, n = 6–8. Scale bars: 500 nm in TEM images; 20 μm in fluorescent and light microscopic images.

To visualize the consequences of sepiapterin treatment on the endothelial glycocalyx, we quantified the binding of cationic ferritin to the negatively-charged glycocalyx. As shown in Fig 4, diabetic mice displayed decreased endothelial coverage (40.7 ± 7.5%), as compared to non-diabetic apoE KO mice (83.6 ± 8.3%, P<0.05; Fig 4A and 4B [19]). Interestingly, treatment with sepiapterin seems to lead to a partial restoration of the glycocalyx, based on the observation that some glomeruli in non-diabetic mice display cationic ferritin coverage, whereas other glomeruli do not. This phenomenon resulted in high variability in glycocalyx-coverage data (61.6 ± 25.8%, P<0.1).

In diabetes, various factors can lead to disruption of the glycocalyx [29]. Heparanase-mediated degradation of heparan sulphate represents one of the most widely recognized causes of glycocalyx perturbation. In particular locally active heparanase plays a critical role in the development of diabetic nephropathy in mice [30]. We previously confirmed enhanced glomerular heparanase protein expression in diabetic apoE mice, compared to non-diabetic apoE KO mice (39.3 ± 10.8% vs. 13.1 ± 9.2%, P<0.01; Fig 4A and 4C). A notable observation is that sepiapterin treatment did not lead to a reduction in the expression levels of this enzyme (36.1 ± 7.4.8%), nor in the heparanase activator cathepsin L (30.2 ± 12.5% vs. 27.3 ± 11.3 for non-treated diabetic mice). This suggests that heparanase is continuously activated by immunocyte derived cathepsin L despite treatment with sepiapterin, which could potentially explain that restoration of glycocalyx coverage by sepiapterin in diabetic nephropathy was only partial.

Despite the observed partial restoration of the luminal glycocalyx and possible regulatory effect of eNOS on heparanase [31], increasing NO bioavailability with sepiapterin was not capable to restore endothelial function.

## Discussion

Endothelial dysfunction plays a critical role in the pathogenesis of diabetes. Diabetes is associated with loss of glomerular barrier function. Activation of extracellular heparanase by inflammatory cells or injured epithelial cells was shown to be of critical importance to cause loss of the glomerular endothelial glycocalyx and proteinuria [30, 32]. Nitric oxide (NO) through intracellular s-nitrosylation is a key modifier of cellular function and to ensure cellular quiescence and tissue homeostasis. The three NO synthases (eNOS, iNOS and nNOS) are however complex oxidoreductases with the potential to produce NO as well as reactive oxygen species [33]. Specifically, for the production of NO they require the cofactor tetrahydrobiopterin (BH_4_). Diabetes has been shown to result in loss of BH_4_ availability and uncoupling of NOS [7, 34].

The present study shows that improving the NO producing capacity at the tissue level in the kidney does not result in reduction of glomerular heparanase activity or improvement of glomerular barrier function. Our observations add several new insights about 1] NO measurements, 2] NO sources in different diabetes models, and 3] the glomerular endothelial glycocalyx:

1. When measuring NO availability at the tissue level using state-of-the-art spin trapping technique (EPR) that allows for quantitative NO measurements, diabetes perse is not associated with overall reduction in NO. In fact, NO availability was increased in the kidney and heart upon diabetes, while liver NO was decreased, despite elevated levels of the endogenous NOS inhibitor asymmetric dimethylarginine (ADMA). Reports on NO availability in diabetes have varied from decreased to increased, pointing to the variation in models and the often indirect measurements of NO activity. Given the complex nature of *in vivo* NO radical detection, surrogate markers for free NO levels have oftentimes been utilized as a readout, including plasma NOx levels, nitrate, nitrosothiols or nitrosylated heme. In this context, we note that previous reports of enhanced NO production in diabetes [8] were based on the quantification of downstream metabolites of NO, such as NOx or nitrate. Furthermore, previous reports on decreased renal NO production in rat 7–10 days after induction of diabetes are based on decreased urinary nitrite/nitrate excretion [35, 36] or decreased plasma nitrite/nitrate levels at 8 weeks after induction of diabetes [15]. Importantly, these NO metabolites serve as poor indicators of the actual NO free radical levels. This is largely in part due to the fact that enhanced synthesis of NO augments NOx generation, whereas a concomitant increase in oxidative stress or reactive oxygen species (ROS) has been established to trigger a rapid depletion of local NO levels. As such, the detection of MNIC, the formation of which is specific for NO free radicals in biological tissues, represents a more suitable experimental and diagnostic approach for detecting local NO levels [21, 22, 37].
2. Renal NO production in diabetes will be dependent upon different sources including endothelium, macrophages [38] and the tubular system [39]. Chronic uncoupling or inhibition of eNOS has been shown to accelerate kidney disease [40, 41]. Conditions commonly observed in patients with kidney disease, such as hyperglycemia, are characterized by an increase in the generation of advanced glycation end products (AGEs) [42]. AGEs actively promote NO insufficiency by scavenging NO free radicals [13] via pro-inflammatory AGE-specific receptors [43–45], or modification of plasma proteins such as albumin [46]. We recently described that the streptozotocin induced diabetic apoE mouse model, which faithfully recapitulates the renal changes in diabetes, is characterized by macrophage activation, which may explain the increased NO availability. McNeill et al showed in a series of elegant studies that macrophage function is modulated by NOS coupling and BH_4_ availability [4], as was previously also shown by us for endothelial function [3]. The current data show that it is possible to increase NO production further in diabetes by coupling through BH_4_ availability, indicating that uncoupling of NOS enzymes was present in the model. In contrast to the current study, sepiapterin showed renal protective effect in diabetic db/db mice [47] and ZSF_1_ rats, which can possibly be explained by the fact that these models are primarily characterized by insulin resistance. Insulin signaling is coupled to NOS activation and impaired insulin signaling may thus have altered the coupling state of NOS in these models. In our model, mice received small amounts of insulin to keep blood glucose concentrations within reasonable range. Given that insulin stimulates NO release by endothelial cells [48, 49], this could serve as an explanation for the fact that we did not observe overall NO deficiency in our diabetic apoE KO mice.
3. Endothelial cells are covered with a dense layer of proteoglycans and glycosaminoglycans, the endothelial glycocalyx. It is the first barrier of the vascular wall and is vasculoprotective [50] by acting as a permeability barrier [51–53], a mechanosensor [50, 54–57], and by regulating inflammation [58–61]. Loss of endothelial glycocalyx occurs, amongst others, during oxidative stress and inflammation, both present in diabetes. This is related to the induction of glycocalyx degrading enzymes, such as hyaluronidase, heparanase and chondroitinase [62, 63]. Of these, heparanase activity has been suggested to be affected by NO bioavailability [31]. Heparanase-induced glycocalyx degradation leads to albuminuria [1] and increased glomerular heparanase expression was shown to be associated with the development of diabetic nephropathy in humans and mice [30, 64].

Immunocytes have been implicated in activation of extracellular heparanase and degradation of heparan sulfate in the glycocalyx [1]. Whereas multiple cells can secrete heparanase upon activation, including endothelium, podocytes and immunocytes [30], its activation requires the cleavage of a linker protein by the protease cathepsin L [30, 65]. While sepiapterin successfully increased the modulating potential of NO in this model of diabetic nephropathy, immunocytes were not affected, hence glomerular heparanase and cathepsin L were not reduced and glycocalyx properties not restored.

## Conclusion

In conclusion, our data show that overall NO availability at the tissue level is not reduced in diabetes, despite the presence of factors causing endothelial dysfunction, and the presence of increased levels of the endogenous NOS inhibitor ADMA. Targeting uncoupled NOS with the BH_4_ precursor sepiapterin increases NO availability, but does not modify renal glomerular endothelial barrier function.
